# Supplementary material for: Enhancing drought resilience in durum wheat: effect of root architecture and genotypic performance in semi-arid rainfed regions
Source: PeerJ. 2025 Mar 27;13:e19096. doi: 10.7717/peerj.19096 (PMC11955194; doi:10.7717/peerj.19096)
Supplement: Table S1 [file peerj-13-19096-s001.docx]

Table S1. List of the 30 varieties of durum wheat evaluated during the two cropping seasons.

| **Nº** | **Genotype** | | **Abbreviation** | | **Type/Pedigree** | | **Origin** | | **Year of release** | |
| --- | --- | --- | --- | --- | --- | --- | --- | --- | --- | --- |
| 1 | Bidi 17 | | Bid | | Landrace | | Algeria | | 1930 | |
| 2 | Gloire de Montgolfier | | Glo | | Landrace | | Algeria | | 1960 | |
| 3 | Guemgoum R'khem | | Gue | | Landrace | | Algeria | | 1960 | |
| 4 | Hedba 3 | | Hed | | Landrace | | Algeria | | 1921 | |
| 5 | Langlois | | Lan | | Landrace | | Algeria | | 1930 | |
| 6 | Mohammed Ben Bachir | | MBB | | Landrace | | Algeria | | 1930 | |
| 7 | Montpellier | | Mon | | Landrace | | Algeria | | 1965 | |
| 8 | Oued Zenati 368 | | OZ | | Landrace | | Algeria | | 1936 | |
| 9 | Acsad 65 | | Acs | | Gerardo-vz-469/3/Jori-1//Nd-61-130/Leeds | | ACSAD | | 1984 | |
| 10 | Altar 84 | | Alt | | Ruff/Flamingo,mex//Mexicali-75/3/Shearwater | | CYMMIT | | 1984 | |
| 11 | Bousselem | | Bou | | Heider//Martes/Huevos de oro | | ICARDA | | 2007 | |
| 12 | Boutaleb | | Bot | | Hedba 3/Ofanto | | Algeria | | 2013 | |
| 13 | Capeiti | | Cap | | Eiti*6/Senatore-Cappelli | | Italy | | 1940 | |
| 14 | Cirta | | Cir | | Hedba-3/Gerardo-vz-619 | | Algeria | | 2000 | |
| 15 | GTA Dur | | GTA | | Crane/4/Polonicum PI185309//T.glutin enano/2* Tc60/3/Gll | | CIMMYT | | 1972 | |
| 16 | INRAT 69 | | INR | | Mahmoudi/(bd-2777)Kyperounda | | Tunisia | | 1969 | |
| 17 | Korifla | | Kor | | Durum-dwarf-s-15/Crane//Geier | | ICARDA | | 1987 | |
| 18 | Mansourah | | Man | | Bread wheat/MBB | | Algeria | | 2012 | |
| 19 | Massinissa | | Mas | | Ofanto/Bousselem | | Algeria | | 2012 | |
| 20 | Megress | | Mgs | | Ofanto/Waha//MBB | | Algeria | | 2007 | |
| 21 | Mexicali 75 | | Mex | | Gerardo-vz-469/3/Jori(sib)//Nd-61-130/Leeds | | CIMMYT | | 1975 | |
| 22 | Ofanto | | Ofa | | Ademelio/Appulo | | Italy | | 1990 | |
| 23 | Oued El Berd | | OEB | | Gta dur/Ofanto | | Algeria | | 2013 | |
| 24 | Polonicum | | Pol | | Triticum polinicum/Zenati boulette 1953-58 | | France | | 1973 | |
| 25 | Sahell | | Sah | | Cit”s”/4/Tace/4*tc//2*zb/wls/3/aa”s’’/5/Ruff”s”/Albe”s” | | CYMMIT | | 1977 | |
| 26 | Simeto | | Sim | | Capeiti-8/Valnova | | Italy | | 1988 | |
| 27 | Sitifis | | Sit | | Bousselam/Ofanto | | Algeria | | 2011 | |
| 28 | Vitron | | Vit | | Turkey77/3/Jori/Anhinga//Flamingo | | Spain | | 1987 | |
| 29 | Waha | | Wah | | Plc/Ruff//Gta’s/3/Rolette | | ICARDA | | 1986 | |
| 30 | ZB×Fg | | ZBF | | Zb/fg‘‘s’’ lk/3/ko 120/4/Ward cs 10604 | | Algeria | | 1983 | |
| *: Backross | |  | |  | |  | |  | |  |
