# Supplementary material for: Enhancing drought resilience in durum wheat: effect of root architecture and genotypic performance in semi-arid rainfed regions
Source: PeerJ. 2025 Mar 27;13:e19096. doi: 10.7717/peerj.19096 (PMC11955194; doi:10.7717/peerj.19096)
Supplement: Table S2 [file peerj-13-19096-s002.docx]

Table S2. Minimum, maximum, mean comparison and drought reduction (Redu %) for the recorded traits.

|  |  | Drought season | | | | |  | | Wet season | | |  |  |
| --- | --- | --- | --- | --- | --- | --- | --- | --- | --- | --- | --- | --- | --- |
| Traits |  | Min | | Max | | Mean |  | Min | | Max | Mean |  | Redu% |
| PEM |  | 121.43 | 310.71 | | 204.09 ^a^ | |  | 128.57 | | 332.14 | 184.66 ^a^ |  | -10.52 |
| PH (cm) |  | 40.00 | 95.00 | | 65.00 ^b^ | |  | 75.20 | | 143.56 | 97.95 ^a^ |  | 33.64 |
| SNA |  | 150.00 | 335.71 | | 230.54^b^ | |  | 170.00 | | 472.50 | 317.90 ^a^ |  | 27.48 |
| GNS |  | 9.24 | 29.64 | | 19.80 ^b^ | |  | 8.70 | | 40.86 | 28.58 ^a^ |  | 30.72 |
| GNA |  | 2077.35 | 8194.28 | | 4553.71 ^b^ | |  | 2022.60 | | 15323.97 | 9077.36 ^a^ |  | 49.83 |
| DTH (days) |  | 106.00 | 118.00 | | 109.53 ^b^ | |  | 115.00 | | 130.00 | 122.51 ^a^ |  | 10.59 |
| TKW (g) |  | 27.60 | 50.60 | | 35.00 ^b^ | |  | 44.68 | | 68.60 | 53.41 ^a^ |  | 34.47 |
| AGB (Mg ha^-1^) |  | 0.84 | 5.48 | | 3.80 ^b^ | |  | 7.54 | | 28.12 | 15.94 ^a^ |  | 76.14 |
| PGY (Mg ha^-1^) |  | 0.83 | 2.53 | | 1.79 ^b^ | |  | 0.84 | | 8.00 | 5.28 ^a^ |  | 66.10 |
| HI |  | 0.19 | 0.59 | | 0.41 ^a^ | |  | 0.11 | | 0.47 | 0.31 ^b^ |  | -31.79 |
| FLA (cm^2^) |  | 9.39 | 24.55 | | 14.76 ^b^ | |  | 14.22 | | 38.09 | 20.81 ^a^ |  | 29.08 |
| SLW (mg cm^-2^) |  | 7.02 | 9.98 | | 8.29 ^a^ | |  | 3.77 | | 8.57 | 6.96 ^b^ |  | -19.5 |
| LRN |  | 1.00 | 5.00 | | 2.84 ^a^ | |  | 1.00 | | 5.00 | 2.84^a^ |  | 0.06 |
| RWC (%) |  | 72.58 | 92.14 | | 80.83 ^b^ | |  | 77.45 | | 99.79 | 89.67 ^a^ |  | 9.86 |
| CT (°C) |  | 23.00 | 31.00 | | 26.48^b^ | |  | 24.00 | | 38.60 | 28.97^a^ |  | 8.58 |

PEM: Plant emergence per area, PH: Plant height, SNA: Spike number per area, GNS: Grain number per spike, GNA: Grain number per area, DTH: Days to heading, TKW: Thousand kernel weight, AGB: Above ground biomass, PGY: Plot grain yield, HI: Harvest index, FLA: Flag leaf area, SLW: Specific leaf weight, LRN: Leaf rolling at noon, RWC: Relative water content, , CT: Canopy temperature. Different letters indicate significant difference between drought and wet year at 0.05 level of significance.
