# Supplementary material for: Enhancing drought resilience in durum wheat: effect of root architecture and genotypic performance in semi-arid rainfed regions
Source: PeerJ. 2025 Mar 27;13:e19096. doi: 10.7717/peerj.19096 (PMC11955194; doi:10.7717/peerj.19096)
Supplement: Table S3 — Comparison performed considering all genotypes together (cultivars and landraces). [file peerj-13-19096-s003.docx]

Table S3. Comparison of harvested grain yield for drought and non-drought cropping season for 30 durum wheat varieties including cultivars and landraces. Comparison performed considering all genotypes together (cultivars and landraces).

| Varieties | Type | PGY, Mg ha^-1^  (drought) | PGY, Mg ha^-1^  (wet year) |
| --- | --- | --- | --- |
|  |  |  |  |
| **Landraces** |  |  |  |
|  |  |  |  |
| Bidi 17 | Landrace | 1.84 abcdef | 4.71 efghij |
| Gloire de Montgolfier | Landrace | 1.60 cdefg | 4.27 ghijk |
| Guemgoum R'khem | Landrace | 1.47 fg | 3.37 k |
| Hedba 3 | Landrace | 1.82 abcdef | 5.04 defghij |
| Langlois | Landrace | 1.70 abcdef | 3.81 ijk |
| Mohammed Ben Bachir | Landrace | 1.62 bcdefg | 5.20 cdefgh |
| Montpellier | Landrace | 1.50 efg | 5.47 bcdefg |
| Oued Znati 368 | Landrace | 1.98 abcde | 4.09 hijk |
|  |  |  |  |
|  |  |  |  |
| **Cultivars** |  |  |  |
|  |  |  |  |
| Acsad 65 | Cultivar | 1.65 abcdefg | 5.27 cdefgh |
| Altar 84 | Cultivar | 1.70 abcdef | 3.72 jk |
| Bousselem | Cultivar | 1.67 abcdef | 6.15 abcd |
| Boutaleb | Cultivar | 1.90 abcdef | 4.67 efghijk |
| Capeiti | Cultivar | 1.58 defg | 5.43 bcdefg |
| Cirta | Cultivar | 1.95 abcdef | 6.35 abcd |
| GTA Dur | Cultivar | 1.99 abcd | 6.83 a |
| INRAT 69 | Cultivar | 1.17 g | 5.14 cdefghi |
| Korifla | Cultivar | 2.08 abc | 5.67 abcdef |
| Mansourah | Cultivar | 1.77 abcdef | 4.45 fghijk |
| Massinissa | Cultivar | 2.02 abcd | 5.98 abcde |
| Megress | Cultivar | 2.01 abcd | 6.36 abc |
| Mexicali 75 | Cultivar | 2.11 a | 6.65 ab |
| Ofanto | Cultivar | 1.56 defg | 6.11 abcd |
| Oued El Berd | Cultivar | 1.93 abcdef | 4.57 fghijk |
| Polonicum | Cultivar | 2.00 abcd | 5.50 abcdefg |
| Sahel 77 | Cultivar | 1.81 abcdef | 5.17 cdefgh |
| Simeto | Cultivar | 1.62 abcdefg | 5.76 abcdef |
| Sitifis | Cultivar | 2.10 ab | 6.12 abcd |
| Vitron | Cultivar | 2.08 abc | 5.92 abcde |
| Waha | Cultivar | 1.86 abcdef | 5.40 bcdefgh |
| ZB/Fg | Cultivar | 1.73 abcdef | 5.16 cdefgh |

Same letter (s) indicate no significant difference between genotypes.
