# Supplementary material for: Enhancing drought resilience in durum wheat: effect of root architecture and genotypic performance in semi-arid rainfed regions
Source: PeerJ. 2025 Mar 27;13:e19096. doi: 10.7717/peerj.19096 (PMC11955194; doi:10.7717/peerj.19096)
Supplement: Table S4 [file peerj-13-19096-s004.docx]

Table S4. Pearson coefficients for the assessed traits under the cropping season 2016/2017 and 2017/2018, drought (above diagonal) and wet year (below diagonal), respectively.

|  | PEM | PH | GNA | DTH | TKW | AGB | SNA | GNS | PGY | HI | FLA | SLW | RWC | LRN | CT |
| --- | --- | --- | --- | --- | --- | --- | --- | --- | --- | --- | --- | --- | --- | --- | --- |
| PEM | -0.06 | -0.09 | 0.16 | -0.14 | **-0.55** | 0.03 | **0.50** | -0.19 | 0.10 | -0.22 | -0.29 | -0.31 | -0.16 | -0.24 | 0.35 |
| PH (cm) | 0.04 | **0.80** | 0.12 | **0.86** | 0.17 | **0.61** | 0.03 | 0.11 | -0.08 | -0.25 | **0.57** | **-0.48** | **0.49** | 0.28 | -0.25 |
| GNA | 0.11 | **-0.44** | 0.32 | 0.00 | -0.29 | **0.43** | **0.53** | **0.75** | **0.55** | **0.58** | -0.07 | 0.18 | -0.25 | -0.01 | 0.10 |
| DTH (days) | 0.17 | **0.75** | **-0.61** | **0.77** | 0.28 | **0.69** | -0.03 | 0.02 | -0.09 | -0.38 | **0.62** | -0.36 | **0.54** | 0.26 | -0.25 |
| TKW (g) | **-0.42** | -0.05 | -0.26 | -0.07 | **0.59** | 0.15 | -0.32 | -0.08 | 0.04 | 0.22 | 0.22 | 0.12 | 0.16 | 0.19 | 0.02 |
| AGB (Mg ha^-1^) | -0.28 | 0.21 | 0.17 | 0.07 | -0.18 | 0.19 | 0.37 | 0.23 | 0.14 | -0.21 | **0.45** | -0.14 | **0.42** | 0.38 | -0.13 |
| SNA | -0.08 | -0.33 | **0.53** | -0.35 | -0.18 | **0.61** | **0.26** | -0.15 | 0.18 | 0.07 | -0.09 | -0.10 | -0.05 | 0.05 | -0.14 |
| GNS | 0.34 | -0.07 | **0.57** | -0.17 | -0.15 | -0.25 | -0.13 | 0.33 | **0.52** | **0.62** | -0.03 | 0.29 | -0.24 | -0.03 | 0.21 |
| PGY (Mg ha^-1^) | 0.22 | **-0.58** | **0.58** | **-0.49** | -0.08 | 0.16 | **0.55** | 0.33 | 0.39 | **0.54** | -0.13 | 0.10 | -0.24 | -0.06 | 0.10 |
| HI | 0.10 | **-0.64** | **0.60** | **-0.63** | 0.10 | -0.38 | 0.17 | **0.53** | **0.65** | **0.42** | -0.33 | 0.40 | **-0.54** | -0.20 | 0.24 |
| FLA (cm^2^) | 0.01 | **0.52** | -0.15 | 0.31 | 0.11 | -0.03 | -0.24 | 0.11 | -0.18 | -0.17 | **0.55** | -0.36 | **0.66** | **0.64** | **-0.43** |
| SLW (mg cm-^2^) | -0.24 | **-0.61** | 0.21 | **-0.51** | 0.16 | -0.02 | 0.06 | 0.05 | 0.22 | 0.23 | **-0.57** | 0.27 | -0.37 | -0.09 | 0.06 |
| RWC (%) | 0.11 | -0.15 | 0.05 | 0.18 | -0.28 | 0.12 | 0.15 | -0.11 | 0.10 | -0.08 | -0.34 | 0.04 | -0.29 | **0.53** | **-0.59** |
| LRN | 0.23 | 0.20 | -0.17 | 0.05 | 0.20 | -0.15 | -0.24 | 0.09 | 0.01 | 0.01 | 0.22 | -0.03 | -0.20 | 1.00 | -0.38 |
| CT (°C) | 0.06 | 0.14 | -0.11 | 0.12 | 0.05 | 0.01 | -0.02 | 0.00 | -0.19 | -0.01 | 0.20 | -0.30 | -0.01 | 0.09 | 0.15 |

PEM: Plant emergence per area, PH: Plant height, SNA: Spike number per area, GNS: Grain number per spike, GNA: Grain number per area, DTH: Days to heading, TKW: Thousand kernel weight, AGB: Above ground biomass, PGY: Plot grain yield, HI: Harvest index, FLA: Flag leaf area, SLW: Specific leaf weight, LRN: Leaf rolling at noon, RWC: Relative water content, CT: Canopy temperature. Values in bold are different from 0 with a significance level alpha=0.05. Cells in grey are the correlations between drought and wet conditions for the corresponding.
