# Supplementary material for: Enhancing drought resilience in durum wheat: effect of root architecture and genotypic performance in semi-arid rainfed regions
Source: PeerJ. 2025 Mar 27;13:e19096. doi: 10.7717/peerj.19096 (PMC11955194; doi:10.7717/peerj.19096)
Supplement: Figure S1 — The traits measured (initial traits) were subjected to cluster analysis for each season. [file peerj-13-19096-s006.pdf]

Initial traits

Abbreviation	Name
AGB	Above ground biomass
CT	Canopy temperature
DTH	Days to heading
FLA	Flag leaf area
FLDW	Flag leaf dry weight
FLFW	Flag leaf fresh weight
FLL	Flag leaf length
FLW	Flag leaf width
GNA	Grain number per area
GNS	Grain number per spike
HI	Harvest index
LRM	Leaf rooling index at morning
LRN	Leaf rooling index at noon
PEM	Plant emergence
PGY	[Plot grain yield
PH	Plant height
RWC	Relative water content
SLW	Specific leaf weight
SNA	Spike number per area
SpkBio	Spike biomass
StemBio	Stem biomass
SWA	Spike weight per area
TKW	Thousand kernel weight

Cluster analysis

Traits in blue color were selected from each dendrogram

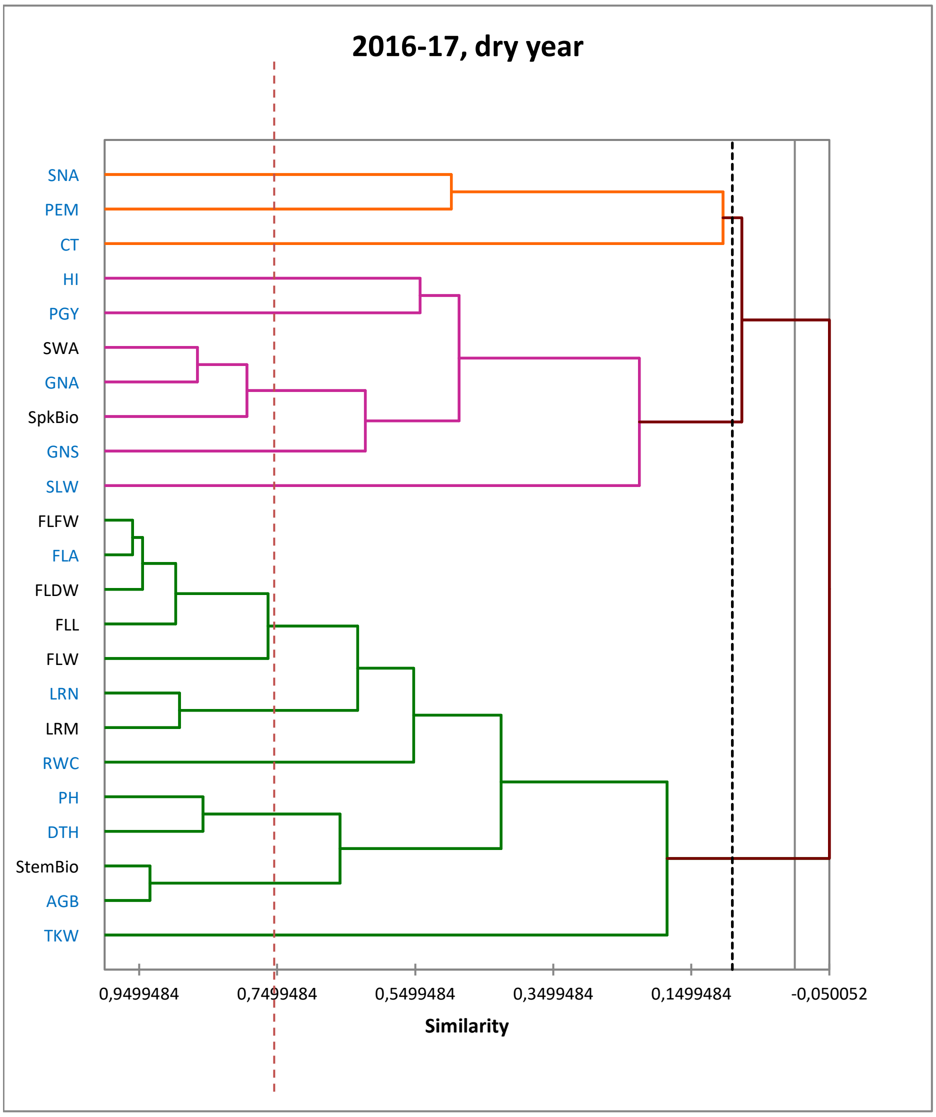

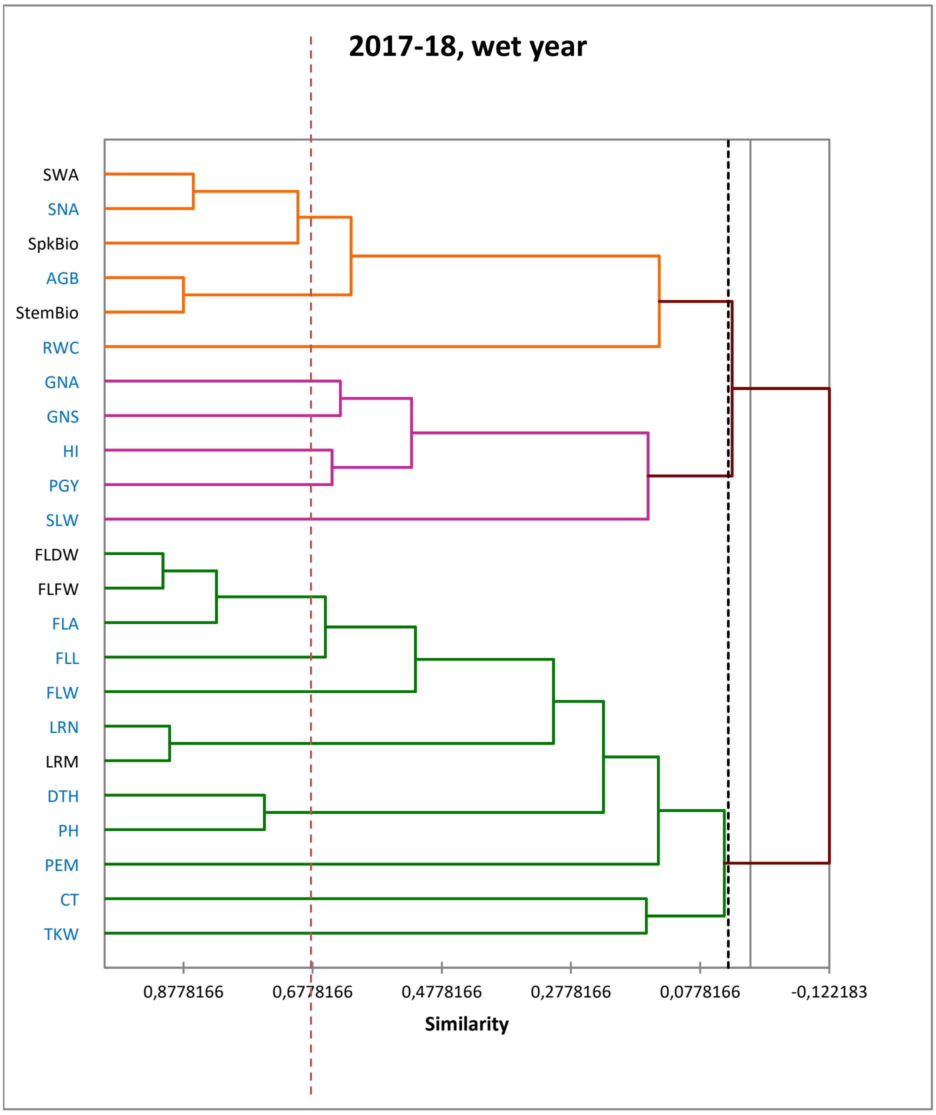
2017-18, wet year
SWA
SNA
SpkBio
AGB
StemBio
RWC
GNA
GNS
HI
PGY
SLW
FLDW
FLFW
FLA
FLL
FLW
LRN
LRM
DTH
PH
PEM
CT
TKW
0,8778166
0,6778166
0,4778166
0,2778166
0,0778166
-0,122183
Similarity

Trait selection

2016-17	2017-18	Intersection
AGB	AGB	AGB
CT	CT	CT
DTH	DTH	DTH
FLA	FLA	FLA
GNA	GNA	GNA
GNS	GNS	GNS
HI	HI	HI
LRN	LRN	LRN
PEM	PEM	PEM
PGY	PGY	PGY
PH	PH	PH
RWC	RWC	RWC
SLW	SLW	SLW
SNA	SNA	SNA
TKW	TKW	TKW
	FLL	
	FLW	
