# Supplementary figures and images for: Enhancing drought resilience in durum wheat: effect of root architecture and genotypic performance in semi-arid rainfed regions
Source: PeerJ. 2025 Mar 27;13:e19096. doi: 10.7717/peerj.19096 (PMC11955194; doi:10.7717/peerj.19096)

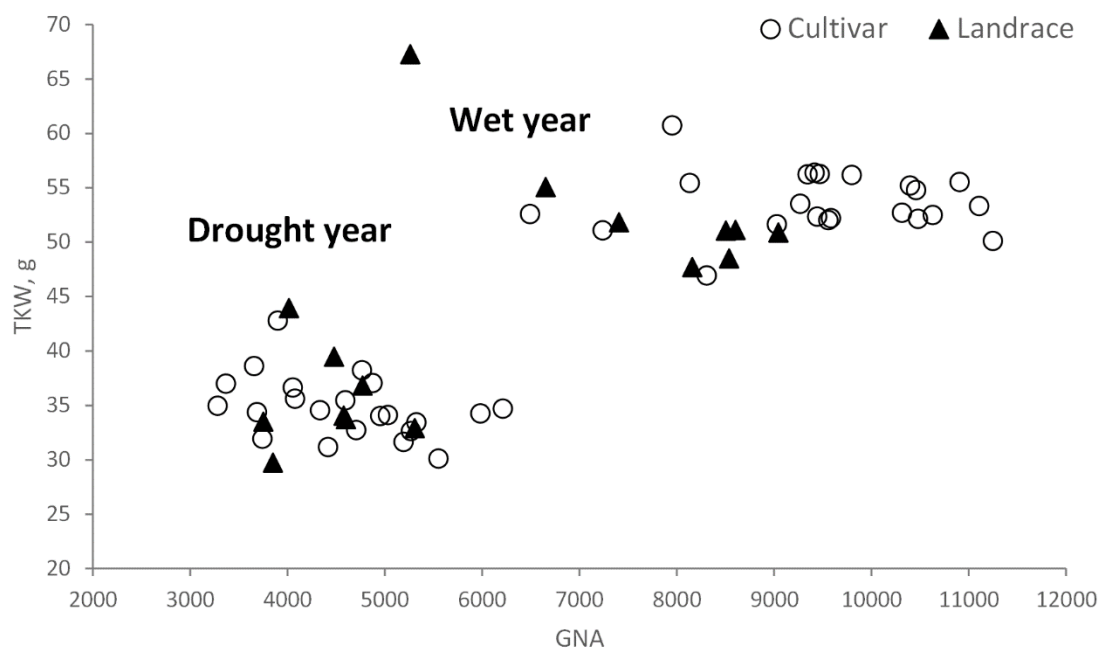

Supplement: Figure S2 [file peerj-13-19096-s007.pdf]
